# Supplementary material for: Maternal ancestry analyses of red tilapia strains based on D-loop sequences of seven tilapia populations
Source: PeerJ. 2019 May 29;7:e7007. doi: 10.7717/peerj.7007 (PMC6545098; doi:10.7717/peerj.7007)
Supplement: Data S1 — Raw sequences exported from the ABI3730XL sequencing instrument applied for data analyses and preparation for Table 1. [file peerj-07-7007-s001.docx]

>DL_1 MH515150

CCGAGCTCTGCCTTCATGCAAATACAATGCATATATGTATTATCACCATTATTTTATATCAAACATATCCTATATATAAATACATACTTTTTTAAAAGACATCCACTGCTCCCCCACATATTTGCCAACAACATTTACAACTAAGAGAAACATAAACCAGTAAATGGAACTTTCCAAAAACATTTCAAAACCACTAAACGACAGTTTAAGACCGAACACAACTCTCATACGTTAAGATATACCAAGTACCCACCATCCTATACTTCCGAATTATTTAATGTAGTAAGAGCCCACCATCAGTTGATTTTTCAATGTTAACGGTTTTTGAAGGTCAAGGACAGTTATTTGTGGGGGTTTCACTAATTGAATTATTCCTGGCATTTGGTTCCTATTTCAGGTCCAATAATTGTTATAATTCCCCATTCTTTCATCGACGCTTGCATAAGTTAATGGTGTTAATACATACTCCTCGTTACCCACCATGCCGGGCGTTCTTTCCAGGGTGTGGGGGGTTTTTTTTTTTTTTTTCCTTTCACTTGACATTTCAGAGTGCATACAGAAAAGACAGACAAGGTTGAACATTTTCCTTGCTTGAACGGAAATAGTATGAATGGTGGTTAGATATTATTAAGAAGAATTGCATAACTGATATCTAGAGCATAAAGTTTAATCAAAATTTTAATTTTCTCCTAATTTTTCTATTAACCTTCGGTTTTTGCGCGCGTAAACCCCCCCTACCCCCCCAAAACTCCTAAGATCTCTAATATTCCTGCAAACCCCCCGGAAACAGGAAAAGCTCTAGAAGTAACTTTCAGCGCTTTAATATATGCATAAAATATTACTTAATGTGTGTATATCTAGTAC

>DL_2 MH515151

CCGAGCTCTGCCTTCATGCAAATACAATGCATATATGTATTATCACCATTATTTTATATCAAACATATCCTATATATAAATACATACTTTTTTAAAAGACATCCACTGCTCCCCCACATATTTGCCAACAACATTTACAACTAAGAGAAACATAAACCAGTAAATGGAACTTTCCAAAAACATTTCAAAACCACTAAACGACAGTTTAAGACCGAACACAACTCTCATACGTTAAGATATACCAAGTACCCACCATCCTATACTTCCGAATTATTTAATGTAGTAAGAGCCCACCATCAGTTGATTTCTCAATGTTAACGGTTCTTGAAGGTCAAGGACAGTTATTCGTGGGGGTTTCACTAATTGAATTATTCCTGGCATCTGGTTCCTATTTCAGGTCCAATAATTGTTATAATTCCCCATTCTTTCATCGACGCTTGCATAAGTTAATGGTGTTAATACATACTCCTCGTTACCCACCATGCCGGGCGTTCTTTCCAGGGTGTGGGGGGTTCTCTTTTTTTTTTTCCTTTCACTTGACATTTCAGAGTGCATACAGAAAAGACAGACAAGGTTGAACATTTTCCTTGCTTGAACGGAAATAGTATGAATGGTGGTTAGATATTATTAAGAAGAATTGCATAACTGATATCTAGAGCATAAAGTTTAATCAAAATTTTAATTTTCTCCTAATTTTTCTATTAACCTTCGGTTTTTGCGCGCGTAAACCCCCCCTACCCCCCCAAAACTCCTAAGATCTCTAATATTCCTGCAAACCCCCCGGAAACAGGAAAAGCTCTAGAAGTAACTTTCAGCGCTTTAATATATGCATAAAATATTACTTAATGTGTGTATATCTAGTAC

>DL_4 MH515153

CCGAGCTCTGCCTTCATGCAAATACAATGCATATATGTATTATCACCATTATTTTATATCAAACATATCCTATATATAAATACATACTTTTTTAAAAGACATCCACTGCTCCCCCACATATTTGCCAACAACATTTACAACTAAGAGAAACATAAACCAGTAAATGGAACTTTCCAAAAACATTTCAAAACCACTAAACGACAGTTTAAGACCGAACACAACTCTCATACGTTAAGATATACCAAGTACCCACCATCCTATACTTCCGAATTATTTAATGTAGTAAGAGCCCACCATCAGTTGATTTCTCAATGTTAACGGTTCTTGAAGGTCAAGGACAGTTATTCGTGGGGGTTTCACTAATTGAATTATTCCTGGCATCTGGTTCCTATTTCAGGTCCAATAATTGTTATAATTCCCCATTCTTTCATCGACGCTTGCATAAGTTAATGGTGTTAATACATACTCCTCGTTACCCACCATGCCGGGCGTTCTTTCCAGGGTGTGGGGGGTTCTCTTTTTTTTTTTCCTTTCACTTGACATTTCAGAGTGCATACAGAAAAGACAGACAAGGTTGAACATTTTCCTTGCTGGAACGGAAATAGTATGAATGGTGGTTAGATATTATTAAGAAGATTTGCATAACTGATATCTAGAGCATAAAGTTTAATCAAAATTTTAATTTTCTCCTAATTTTTCTATTAACCTTCGGTTTTTGCGCGCGTAAACCCCCCCTACCCCCCCAAAACTCCTAAGATCTCTAATATTCCTGCAAACCCCCCGGAAACAGGAAAAGCTCTAGAAGTAACTTTCAGCGCTTTAATATATGCATAAAATATTACTTAATGTGTGTATATCTAGTAC

>DL_5 MH515154

CCGAGCTCTGCCTTCATGCAAATACAATGCATATATGTATTATCACCATTATTTTATATCAAACATATCCTATATATAAATACATACTTTTTTAAAAGACATCCACTGCTCCCCCACATATTTGCCAACAACATTTACAACTAAGAGAAACATAAACCAGTAAATGGAACTTTCCAAAAACATTTCAAAACCACTAAACGACAGTTTAAGACCGAACACAACTCTCATACGTTAAGATATACCAAGTACCCACCATCCTATACTTCCGAATTATTTAATGTAGTAAGAGCCCACCATCAGTTGATTTCTCAATGTTAACGGTTCTTGAAGGTCAAGGACAGTTATTCGTGGGGGTTTCACTAATTGAATTATTCCTGGCATCTGGTTCCTATTTCAGGTCCAATAATTGTTATAATTCCCCATTCTTTCATCGACGCTTGCATAAGTTAATGGTGTTAATACATACTCCTCGTTACCCACCATGCCGGGCGTTCTTTCCAGGGTGTGGGGGGTTCTCTTTTTTTTTTTCCTTTCACTTGACATTTCAGAGTGCATACAGAAAAGACAGACAAGGTTGAACATTTTCTTTGCTTGAACGGAAATAGTATGAATGGTGGTTAGATATTATTAAGAAGAATTGCATAACTGATATCTAGAGCATAAAGTTTAATCAAAATTTTAATTTTCTCCTAATTTTTCTATTAACCTTCGGTTTTTGCGCGCGTAAACCCCCCCTACCCCCCCAAAACTCCTAAGATCTCTAATATTCCTGCAAACCCCCCGGAAACAGGAAAAGCTCTAGAAGTAACTTTCAGCGCTTTAATATATGCATAAAATATTACTTAATGTGTGTATATCTAGTAC

>DL_6 MH515155

CCGAGCTCTGCCTTCATGCAAAACACAGTACATATATGTATTATCACCATTATTTTATTTCAAACATATCCTATATATAAATACATACAACTCTTAAAAAACATACACTGTTTTCCCACATATTTGTCATCAACATCTATAACTAAGAAGAACATAAACCAATAAATGAAATATTCCAATAACAATTAAATACCACTGAACGATAGTTTAAGACCGAACACAACTCTCATACAGTTAAGATATACCAAGTACCCAACATCCTATACTTCTAAATTATTTAATGTAGTAAGAGCCCACCATCAGTTGATTCCTATATGTTAACGGTTCTTGAAGGTCAAGGACAATTATTTGTGGGGGTTTCACTAATTGAATTATTCCTGGCATCTGGTTCCTATTTCAGGTTCAATAATTGTTATAATTCCCCATTCTTTCATCGACGCTTGCATAAGTTAATGGTGTCAATACATACTCCTCATTACCCAACATGCCGGGCGTTCTTTCCAGAGGATAGGGGGTTTCTCTTTTTTTTTTCCCTTTCACTGGGCATCTCAGAGTGCATACAGAAATGACAGACAAGGTTGAACATTTTCCTTGCTGGAAGGTAAATAGTATGAATGATAGAAGACATTGATAGAAGTATTGCATAACTGATATCTAGAGCATAAAGTTCAATCAAATATTTCAATTTTCTCCTAATTTTTCTATTATTCTTCGGTTTTTTCGCGCGTAAACCCCCCCTACCCCCCCAAAACTCCTAAGATCTCTAATACTCCTGCAAACCCCCCGGAAACAGGAAAAGCTCTAGAAGTGACTTTTAGCGCTTTAGTTTATGCATGTTATATTACTTAATGTGTGTATATGCAGTAT

>DL_7 MH515156

CCGAGCTCTGCCTTCATGCAAATACAATGCATATATGTATTATCACCATTATTTTATATCAAACATATCCTATATATAAATACATACTTTTTTAAAAGACATCCACTGCTCCCCCACATATTTGCCAACAACATTTACAACTAAGAGAAACATAAACCAGTAAATGGAACTTTCCAAAAACATTTCAAAACCACTAAACGACAGTTTAAGACCGAACACAACTCTCATACGTTAAGATATACCAAGTACCCACCATCCTATACTTCCGAATTATTTAATGTAGTAAGAGCCCACCATCAGTTGATTTCTCAATGTTAACGGTTCTTGAAGGTCAAGGACAGTTATTCGTGGGGGTTTCACTAATTGAATTATTCCTGGCATCTGGTTCCTATTTCAGGTCCAATAATTGTTATAATTCCCCATTCTTTCATCGACGCTTGCATAAGTTAATGGTGTTAATACATACTCCTCGTTACCCACCATGCCGGGCGTTCTTTCCAGGGTGTGGGGGGTTCTCTTTTTTTTTTTCCTTTCACTTGACATTTCAAAGTGCATACAGAAAAGACAGACAAGGTTGAACATTTTCCTTGCTTGAACGGAAATAGTATGAATGGTGGTTAGATATTATTAAGAAGAATTGCATAACTGATATCTAGAGCATAAAGTTTAATCAAAATTTTAATTTTCTCCTAATTTTTCTATTAACCTTCGGTTTTTGCGCGCGTAAACCCCCCCTACCCCCCCAAAACTCCTAAGATCTCTAATATTCCTGCAAACCCCCCGGAAACAGGAAAAGCTCTAGAAGTAACTTTCAGCGCTTTAATATATGCATAAAATATTACTTAATGTGTGTATATCTAGTAC

>DL_8 MH515157

CCGAGCTCTGCCTTCATGCAAATACAATGCATATATGTATTATCACCATTATTTTATATCAAACATATCCTATATATAAATACATACTTTTTTAAAAGACATCCACTGCTCCCCCACATATTTGCCAACAACATTTACAACTAAGAGAAACATAAACCAGTAAATGGAACTTTCCAAAAACATTTCAAAACCACTAAACGACAGTTTAAGACCGAACACAACTCTCATACGTTAAGATATACCAAGTACCCACCATCCTATACTTCCGAATTATTTAATGTAGTAAGAGCCCACCATCAGTTGATTTCTCAATGTTAACGGTTCTTGAAGGTCAAGGACAGTTATTCGTGGGGGTTTCACTAATTGAATTATTCCTGGCATCTGGTTCCTATTTCAGGTCCAATAATTGTTATAATTCCCCATTCTTTCATCGACGCTTGCATAAGTTAATGGTGTTAATACATACTCCTCGTTACCCACCATGCCGGGCGTTCTTTCCAGGGTGTGGGGGGTTCTCTTTTTTTTTTTCCTTTCACTTGACATTTCAGAGTGCATACAGAAAAGACAGACAAGGTTGAACATTTTCCTTGCTTGAACGGAAATAGTATGAATGGTGGTTAGATATTATTAAGAAGATTTGCATAATTGATATCTAGAGCATAAAGTTTAATCAAAATTTAATTTTCTCCTAATTTTTCTATTAACCTTCGGTTTTTGCGCGCGTAAACCCCCCCTACCCCCCCAAAACTCCTAAGATCTCTAATATTCCTGCAAACCCCCCGGAAACAGGAAAAGCTCTAGAAGTAACTTTCAGCGCTTTAATATATGCATAAAATATTACTTAATGTGTGTATATCTAGTAC

>DL_9 MH515158

CCGAGCTCTGCCTTCATGCAAAACACAGTACATATATGTATTATCACCATTATTTTATTTCAAACATATCCTATATATAAATACATACAACTCTTAAAAAACATACACTGTTTTCCCACATATTTGTCATCAACATCTATAACTAAGAAGAACATAAACCAATAAATGAAATATTCCAATAACAATTAAATACCACTGAACGATAGTTTAAGACCGAACACAACTCTCATACAGTTAAGATATACCAAGTACCCAACATCCTATACTTCTAAATTATTTAATGTAGTAAGAGCCCACCATCAGTTGATTCCTATATGTTAACGGTTCTTGAAGGTCAAGGACAATTATTTGTGGGGGTTTCACTAATTGAATTATTCCTGGCATCTGGTTCCTATTTCAGGTTCAATAATTGTTATAATTCCCCATTCTTTCATCGACGCTTGCATAAGTTAATGGTGTCAATACATACTCCTCATTACCCAACATGCCGGGCGTTCTTTCCAGAGGATAGGGGGTTTCTCTTTTTTTTTTTCCTTTCACTTGGCATCTCAAAGTGCATACAGAAATGACAGACAAGGTTGAACATTTTCCTTGCTTGAAGGTAAATAGTATGAATGATAGAAGACATTGATAGAAGTATTGCATAACTGATATCTAGAGCATAAAGTTCAATCAAATATTTCAATTTTCTCCTAATTTTCTATTATTCTTCGGTTTTTTCGCGCGTAAACCCCCCCTACCCCCCCAAAACTCCTAAGATCTCTAATACTCCTGCAAACCCCCCGGAAACAGGAAAAGCTCTAGAAGTGACTTTTAGCGCTTTAGTTTATGCATGTTATATTACTTAATGTGTGTATATGCAGTAT

>DL_10 MH515159

CCGAGCTCTGCCTTCATGCAAATACAATGCATATATGTATTATCACCATTATTTTATATCAAACATATCCTATATATAAATACATACTTTTTTAAAAGACATCCACTGCTCCCCCACATATTTGCCAACAACATTTACAACTAAGAGAAACATAAACCAGTAAATGGAACTTTCCAAAAACATTTCAAAACCACTAAACGACAGTTTAAGACCGAACACAACTCTCATACGTTAAGATATACCAAGTACCCACCATCCTATACTTCCGAATTATTTAATGTAGTAAGAGCCCACCATCAGTTGATTTCTCAATGTTAACGGTTCTTGAAGGTCAAGGACAGTTATTCGTGGGGGTTTCACTAATTGAATTATTCCTGGCATCTGGTTCCTATTTCAGGTCCAATAATTGTTATAATTCCCCATTCTTTCATCGACGCTTGCATAAGTTAATGGTGTTAATACATACTCCTCGTTACCCACCATGCCGGGCGTTCTTTCCAGGGTGTGGGGGGTTCTCTTTTTTTTTTTCTTTTCACTTGACATTTCAGAGTGCATACAGAAAAGACAGACAAGGTTGAACATTTTCCTTGCTTGAACGGAAATAGTATGAATGGTGGTTAGATATTATTAAGAAGAATTGCATAACTGATATCTAGAGCATAAAGTTTAATCAAAATTTTAATTTTCTCCTAATTTTTCTATTAACCTTCGGTTTTTGCGCGCGTAAACCCCCCCTACCCCCCCAAAACTCCTAAGATCTCTAATATTCCTGCAAACCCCCCGGAAACAGGAAAAGCTCTAGAAGTAACTTTCAGCGCTTTAATATATGCATAAAATATTACTTAATGTGTGTATATCTAGTAC

>DL_11 MH515160

CCGAGCTCTGCCTTCATGCAAATACAATGCATATATGTATTATCACCATTATTTTATATCAAACATATCCTATATATAAATACATACTTTTTTAAAAGACATCCACTGCTCCCCCACATATTTGCCAACAACATTTACAACTAAGAGAAACATAAACCAGTAAATGGAACTTTCCAAAAACATTTCAAAACCACTAAACGACAGTTTAAGACCGAACACAACTCTCATACGTTAAGATATACCAAGTACCCACCATCCTATACTTCCGAATTATTTAATGTAGTAAGAGCCCACCATCAGTTGATTTCTCAATGTTAACGGTTCTTGAAGGTCAAGGACAGTTATTCGTGGGGGTTTCACTAATTGAATTATTCCTGGCATCTGGTTCCTATTTCAGGTCCAATAATTGTTATAATTCCCCATTCTTTCATCGACGCTTGCATAAGTTAATGGTGTTAATACATACTCCTCGTTACCCACCATGCCGGGCGTTCTTTCCAGGGTGTGGGGGGTTCTCTTTTTTTTTTCCTTTTCACTTGACATTTCAGAGTGCATACAGAAAAGACAGACAAGGTTGAACATTTTCCTTGCTTGAACGGAAATAGTATGAATGGTGGTTAGATATTATTAAGAAGAATTGCATAACTGATATCTAGAGCATAAAGTTTAATCAAAATTTTAATTTTCTCCTAATTTTTCTATTAACCTTCGGTTTTTGCGCGCGTAAACCCCCCCTACCCCCCCAAAACTCCTAAGATCTCTAATATTCCTGCAAACCCCCCGGAAACAGGAAAAGCTCTAGAAGTAACTTTCAGCGCTTTAATATATGCATAAAATATTACTTAATGTGTGTATATCTAGTAC

>DL_12 MH515161

CCGAGCTCTGCCTTCATGCAAAACACAGTACATATATGTATTATCACCATTATTTTATTTCAAACATATCCTATATATAAATACATACAACTCTTAAAAAACATACACTGTTTTCCCACATATTTGTCATCAACATCTATAACTAAGAAGAACATAAACCAATAAATGAAATATTCCAATAACAATTAAATACCACTGAACGATAGTTTAAGACCGAACACAACTCTCATACAGTTAAGATATACCAAGTACCCAACATCCTATACTTCTAAATTATTTAATGTAGTAAGAGCCCACCATCAGTTGATTCCTATATGTTAACGGTTCTTGAAGGTCAAGGACAATTATTTGTGGGGGTTTCACTAATTGAATTATTCCTGGCATCTGGTTCCTATTTCAGGTTCAATAATTGTTATAATTCCCCATTCTTTCATCGACGCTTGCATAAGTTAATGGTGTCAATACATACTCCTCATTACCCAACATGCCGGGCGTTCTTTCCAGAGGATAGGGGGTTTCTCTTTTTTTTTTTCCTTTCACTGGGCATCTCAAAGTGCATACAGAAATGACAGACAAGGTTGAACATTTTCCTTGCTTGAAGGTAAATAGTATGAATGATAGAAGACATTGATAGAAGTATTGCATAACTGATATCTAGAGCATAAAGTTCAATCAAATATTTCAATTTTCTCCTAATTTTTCTATTATTCTTCGGTTTTTTCGCGCGTAAACCCCCCCTACCCCCCCAAAACTCCTAAGATCTCTAATACTCCTGCAAACCCCCCGGAAACAGGAAAAGCTCTAGAAGTGACTTTTAGCGCTTTAGTTTATGCATGTTATATTACTTAATGTGTGTATATGCAGTAT

>DL_13 MH515162

CCGAGGCTCTGCCTTCATGCAAAATACAATGCATATATGTATTATCACCATTATTTTATATCAAACATATCCTATATATAAAATACATACTTTTTTAAAAGACATCACTGCTCCCCCACATATTTGCCAACAACATTTACAACTAAGAGAAACATAAACCAGTAAATGGAACTTTCCAAAAACATTTCAAAACCACTAAACGACAGTTTAAGACCGAACACAACTCTCATACGTTAAGATATACCAAGTACCCACCATCCTATACTTCCGAATTATTTAATGTAGTAAGAGCCCACCATCAGTTGATTTCTCAATGTTAACGGTTCTTGAAGGTCAAGGACAGTTATTTGTGGGGGTTTCACTAATTGAATTATTCCTGGCATTTGGTTCCTATTTCAGGTCCAATAATTGTTATAATTCCCCATTCTTTCATTGACGCTTGCATAAGTTAATGGTGTTAATACATACTCCTCGTTACCCACCATGCCGGGCGTTCTTTCCAGGGTGTGGGGGGTTTTCTTTTTTTTTTTCCTTTCACTTGACATTTCAGAGTGCATACAGAAAAGACAGACAAGGTTGAACATTTTCCTTGCTTGAACGGAAATAGTATGAATGGTGGTTAGATATTATTAAGAAGAATTGCATAACTGATATCTAGAGCATAAAGTTTAATCAAAATTTTAATTTTCTCCTAATTTTTCTATTAACCTTCGGTTTTTGCGCGCGTAAACCCCCCCTACCCCCCCAAAACTCCTAAGATCTCTAATATTCCTGCAAACCCCCCGGAAACAGGAAAAGCTCTAGAAGTAACTTTCAGCGCTTTAATATATGCATAAAATATTACTTAATGTGTGTATATCTAGTAC

>DL_14 MH515163

CCGAGCTCTGCCCTCATGCAAATACAATGCATATATGTATTATCACCATTATTTTATATCAAACCATATCCTATATATAAAATACATACTTTTTAAAAGACATCCACTGCTCCCCCCACATATTTGCCAACAACATTTACAACTAAGAGAAACATAAACCAGTAAATGGAACTTTCCAAAAACATTTCAAAACCACTAAACGACAGTTTAAGACCGAACACAACTCTCATACGTTAAGATATACCAAGTACCCACCATCCTATACTTCCGGATTATTTAATGTAGTAAGAGCCCACCATCAGTTGATTTCTCCATGTTAACGGTTCTTGAAGGTCAAGGACAGTTATTTGTGGGGGTTTCACTAATTGAATTATTCCTGGCATTTGGTTCCTATTTCAGGTCCAATAATTGTTATAATTCCCCATTCTTTCATCGACGCTTGCATAAGTTAATGGTGTTAATACATACTCCTCGTTACCCACCATGCCGGGCGTTCTTTCCAGGGTGTGGGGGGTTTTCTTTTTTTTTTTCCTTTCACTTGACATTTCAGAGTGCATACAGAAAAGACAGACAAGGTTGAACATTTTCCTTGCTTGAACGGAAATAGTATGAATGGTGGTTAGATATTATTAAGAAGAATTGCATAACTGATATCTAGAGCATAAAGTTTAATCAAAATTTTAATTTTCTCCTAATTTTTCTATTAACCTTCGGTTTTTGCGCGCGTAAACCCCCCCTACCCCCCCAAAACTCCTAAGATCTCTAATATTCCTGCAAACCCCCCGGAAACAGGAAAAGCTCTAGAAGTAACTTTCAGCGCTTTAATATATGCATAAAATATTACTTAATGTGTGTATATCTAGTAC

>DL_15 MH515164

CCGAGCTCTGCCTTCATGCAAATACAATGCATATATGTATTATCACCATTATTTTATATCAAACATATCCTATATATAAATACATACTTTTTTAAAAGACATCCACTGCTCCCCCACATATTTGCCAACAACATTTACAACTAAGAGAAACATAAACCAGTAAATGGAACTTTCCAAAAACATTTCAAAACCACTAAACGACAGTTTAAGACCGAACACAACTCTCATACGTTAAGATATACCAAGTACCCACCATCCTATACTTCCGAATTATTTAATGTAGTAAGAGCCCACCATCAGTTGATTTCTCAATGTTAACGGTTCTTGAAGGTCAAGGACAGTTATTCGTGGGGGTTTCACTAATTGAATTATTCCTGGCATCTGGTTCCTATTTCAGGTCCAATAATTGTTATAATTCCCCATTCTTTCATCGACGCTTGCATAAGTTAATGGTGTTAATACATACTCCTCGTTACCCACCATGCCGGGCGTTCTTTCCAGGGTGTGGGGGGTTCTCTTTTTTTTTTTCCTTTCACTTGACATTTCAAGGTGCATACAGAAAAGACAGACAAGGTTGAACATTTTCCTTGCTTGAACGGAAATAGTATGAATGGTGGTTAGATATTATTAAGAAGAATTGCATAACTGATATCTAGAGCATAAAGTTTAATCAAAATTTTAATTTTCTCCTAATTTTTCTATTAACCTTCGGTTTTTGCGCGCGTAAACCCCCCCTACCCCCCCAAAACTCCTAAGATCTCTAATATTCCTGCAAACCCCCCGGAAACAGGAAAAGCTCTAGAAGTAACTTTCAGCGCTTTAATATATGCATAAAATATTACTTAATGTGTGTATATCTAGTAC

>DL_16 MH515165

GCCGAGCTCTGCCTTCATGCAAATACAATGCATATATGTATTATCACCATTATTTTATATCAAACATATCCTATATATAAATACATACTTTTTTAAAAGACATCCACTGCTCCCCCACATATTTGCCAACAACATTTACAACTAAGAGAAACATAAACCAGTAAATGGAACTTTCCAAAAACATTTCAAAACCACTAAACGACAGTTTAAGACCGAACACAACTCTCATACGTTAAGATATACCAAGTACCCACCATCCTATACTTCCGAATTATTTAATGTAGTAAGAGCCCACCATCAGTTGATTTCTCAATGTTAACGGTTCTTGAAGGTCAAGGACAGTTATTCGTGGGGGTTTCACTAATTGAATTATTCCTGGCATCTGGTTCCTATTTCAGGTCCAATAATTGTTATAATTCCCCATTCTTTCATCGACGCTTGCATAAGTTAATGGTGTTAATACATACTCCTCGTTACCCACCATGCCGGGCGTTCTTTCCAGGGTGTGGGGGGTTCTCTTTTTTTTTTTCCTTTCACTTGACATTTCAAAGTGCATACAGAAAAGACAGACAAGGTTGAACATTTTCCTTGCTTGAACGGAAATAGTATGAATGGTGGTTAGATATTATTAAGAAGAATTGCATAACTGATATCTAGAGCATAAAGTTTAATCAAAATTTTAATTTTCTCCTAATTTTTCTATTAACCTTCGGTTTTTGCGCGCGTAAACCCCCCCTACCCCCCCAAAACTCCTAAGATCTCTAATATTCCTGCAAACCCCCCGGAAACAGGAAAAGCTCTAGAAGTAACTTTCAGCGCTTTAATATATGCATAAAATATTACTTAATGTGTGTATATCTAGTAC

>DL_17 MH515166

CCGAGCTCTGCCTTCATGCAAATACAATGCATATATGTATTATCACCATTATTTTATATCAAACATATCCTATATATAAATACATACTTTTTTAAAAGACATCCACTGCTCCCCCACATATTTGCCAACAACATTTACAACTAAGAGAAACATAAACCAGTAAATGGAACTTTCCAAAAACATTTCAAAACCACTAAACGACAGTTTAAGACCGAACACAACTCTCATACGTTAAGATATACCAAGTACCCACCATCCTATACTTCCGAATTATTTAATGTAGTAAGAGCCCACCATCAGTTGATTTCTCAATGTTAACGGTTCTTGAAGGTCAAGGACAGTTATTCGTGGGGGTTTCACTAATTGAATTATTCCTGGCATCTGGTTCCTATTTCAGGTCCAATAATTGTTATAATTCCCCATTCTTTCATCGACGCTTGCATAAGTTAATGGTGTTAATACATACTCCTCGTTACCCACCATGCCGGGCGTTCTTTCCAGGGTGTGGGGGGTTCTCTTTTTTTTTTTCCTTTCACTTGACATTTCAGAGTGCATACAGAAAAGACAGACAAGGTTGAACATTTTCCTTGCTTGAACGGAAATAGTATGAATGGTGGTTAGATATTATTAAGAAGAATTGCATAACTGATATCTAGAGCATAAAGTTTAATCAAAATTTTAATTTTCCCCTAATTTTTCTATTAACCTTCGGTTTTTGCGCGCGTAAACCCCCCCTACCCCCCCAAAACTCCTAAGATCTCTAATATTCCTGCAAACCCCCCGGAAACAGGAAAAGCTCTAGAAGTAACTTTCAGCGCTTTAATATATGCATAAAATATTACTTAATGTGTGTATATCTAGTAC

>DL_18 MH515167

CCGAGCTCTGCCTTCATGCAAAACACAGTACATATATGTATTATCACCATTATTTTATTTCAAACATATCCTATATATAAATACATACAACTCTTAAAAAACATACACTGTTTTCCCACATATTTGTCATCAACATCTATAACTAAGAAGAACATAAACCAATAAATGAAATATTCCAATAACAATTAAATACCACTGAACGATAGTTTAAGACCGAACACAACTCTCATACAGTTAAGATATACCAAGTACCCAACATCCTATACTTCTAAATTATTTAATGTAGTAAGAGCCCACCATCAGTTGATTCCTATATGTTAACGGTTCTTGAAGGTCAAGGACAATTATTTGTGGGGGTTTCACTAATTGAATTATTCCTGGCATCTGGTTCCTATTTCAGGTTCAATAATTGTTATAATTCCCCATTCTTTCATCGACGCTTGCATAAGTTAATGGTGTCAATACATACTCCTCATTACCCAACATGCCGGGCGTTCTTTCCAGAGGATAGGGGGTTTCTCTTTTTTTTTTTCCTTTCACTTGGCATCTCAAAGTGCATACAGAAATGACAGACAAGGTTGAACATTTTCCTTGCTTGAAGGTAAATAGTATGAATGATAGAAGACATTGATAGAAGTATGGCATAACTGATATCTAGAGCATAAAGTTCAATCAAATATTTCAATTTTCTCCTAATTTTTCTATTATTCTTCGGTTTTTTCGCGCGTAAACCCCCCCTACCCCCCCAAAACTCCTAAGATCTCTAATACTCCTGCAAACCCCCCGGAAACAGGAAAAGCTCTAGAAGTGACTTTTAGCGCTTTAGTTTATGCATGTTATATTACTTAATGTGTGTATATGCAGTAT

>DL_19 MH515168

CCGAGCTCTGCCTTCATGCAAAACACAGTACATATATGTATTATCACCATTATTTTATTTCAAACATATCCTATATATAAATACATACAACTCTTAAAAAACATACACTGTTTTCCCACATATTTGTCATCAACATCTATAACTAAGAAGAACATAAACCAATAAATGAAATATTCCAATAACAATTAAATACCACTGAACGATAGTTTAAGACCGAACACAACTCTCATACAGTTAAGATATACCAAGTACCCAACATCCTATACTTCTAAATTATTTAATGTAGTAAGAGCCCACCATCAGTTGATTCCTATATGTTAACGGTTCTTGAAGGTCAAGGACAATTATTTGTGGGGGTTTCACTAATTGAATTATTCCTGGCATCTGGTTCCTATTTCAGGTTCAATAATTGTTATAATTCCCCATTCTTTCATCGACGCTTGCATAAGTTAATGGTGTCAATACATACTCCTCATTACCCAACATGCCGGGCGTTCTTTCCAGAGGATAGGGGGTTTCTCTTTTTTTTTTTCCTTTCACTTGGCATCTCAAAGTGCATACAGAAATGACAGACAAGGTTGAACATTTTCCTTGCTTGAAGGTAAATAGTATGAATGATAGAAGACATTGATAGAAGTATTGCATAACTGATATCTAGAGCATAAAGTTCAATCAAATATTTCAATTTTCTCCTAATTTTTCTATTATTCTTCGGTTTTTTCGCGCGTAAACCCCCCCTACCCCCCCAAAACTCCTAAGATCTCTAATACTCCTGCAAACCCCCCGGAAACAGGAAAAGCTCTAGAAGTGACTTTTAGCGCTTTAGTTTATGCATGTTATATTACTTAATGTGTGTATATGCAGTAT

>DL_20 MH515169

CCGAGCTCTGCCTTCATGCAAATACAATGCATATATGTATTATCACCATTATTTTATATCAAACATATCCTATATATAAATACATACTTTTTTAAAAGACATCCACTGCTCCCCCACATATTTGCCAACAACATTTACAACTAAGAGAAACATAAACCAGTAAATGGAACTTTCCAAAAACATTTCAAAACCACTAAACGACAGTTTAAGACCGAACACAACTCTCATACGTTAAGATATACCAAGTACCCACCATCCTATACTTCCGAATTATTTAATGTAGTAAGAGCCCACCATCAGTTGATTTCTCAATGTTAACGGTTCTTGAAGGTCAAGGACAGTTATTCGTGGGGGTTTCACTAATTGAATTATTCCTGGCATCTGGTTCCTATTTCAGGTCCAATAATTGTTATAATTCCCCATTCTTTCATCGACGCTTGCATAAGTTAATGGTGTTAATACATACTCCTCGTTACCCACCATGCCGGGCGTTCTTTCCAGGGTGTGGGGGGTTCTCTTTTTTTTTTTCCTTTCACTTGACATTTCAAAGTGCATACAGAAAAGACAGACAAGGTTGAACATTTTCCTTGCTTGAACGGAAATAGTATGAATGGTGGTTAGATATTATTAAGAAGAATTGCATAACTGATATCTAGAGCATAAAGTTTAATCAAAATTTTAATTTTCTCCTAATTTTTCTATTAACCTTCGGTTTTTGCGCGCGTAAACCTCCCCTACCCCCCCAAAACTCCTAAGATCTCTAATATTCCTGCAAACCCCCCGGAAACAGGAAAAGCTCTAGAAGTAACTTTCAGCGCTTTAATATATGCATAAAATATTACTTAATGTGTGTATATCTAGTAC

>DL_21 MH515170

CCGAGCTCTGCCTTCATGCAAATACAATGCATATATGTATTATCACCATTATTTTATATCAAACATATCCTATATATAAATACATACTTTTTTAAAAGACATCCACTGCTCCCCCACATATTTGCCAACAACATTTACAACTAAGAGAAACATAAACCAGTAAATGGAACTTTCCAAAAACATTTCAAAACCACTAAACGACAGTTTAAGACCGAACACAACTCTCATACGTTAAGATATACCAAGTACCCACCATCCTATACTTCCGAATTATTTAATGTAGTAAGAGCCCACCATCAGTTGATTTCTCAATGTTAACGGTTCTTGAAGGTCAAGGACAGTTATTCGTGGGGGTTTCACTAATTGAATTATTCCTGGCATCTGGTTCCTATTTCAGGTCCAATAATTGTTATAATTCCCCATTCTTTCATCGACGCTTGCATAAGTTAATGGTGTTAATACATACTCCTCGTTACCCACCATGCCGGGCGTTCTTTCCAGGGTGTGGGGGGTTCTCTTTTTTTTTTTCCTTTCACTTGACATTTCAAAGTGCATACAGAAAAGACAGACAAGGTTGAACATTTTCCTTGCTTGAACGAAAATAGTATGAATGGTGGTTAGATATTATTAAGAAGAATTGCATAACTGATATCTAGAGCATAAAGTTTAATCAAAATTTTAATTTTCTCCTAATTTTTCTATTAACCTTCGGTTTTTGCGCGCGTAAACCCCCCCTACCCCCCCAAAACTCCTAAGATCTCTAATATTCCTGCAAACCCCCCGGAAACAGGAAAAGCTCTAGAAGTAACTTTCAGCGCTTTAATATATGCATAAAATATTACTTAATGTGTGTATATCTAGTAC

>DL_22 MH515171

CCGAGCTCTGCCTTCATGCAAAACACAGTACATATATGTATTATCACCATTATTTTATTTCAAACATATCCTATATATAAATACATACAACTCTTAAAAAACATACACTGTTTTCCCACATATTTGTCATCAACATCTATAACTAAGAAGAACATAAACCAATAAATGAAATATTCCAATAACAATTAAATACCACTGAACGATAGTTTAAGACCGAACACAACTCTCATACAGTTAAGATATACCAAGTACCCAACATCCTATACTTCTAAATTATTTAATGTAGTAAGAGCCCACCATCAGTTGATTCCTATATGTTAACGGTTCTTGAAGGTCAAGGACAATTATTTGTGGGGGTTTCACTAATTGAATTATTCCTGGCATCTGGTTCCTATTTCAGGTTCAATAATTGTTATAATTCCCCATTCTTTCATCGACGCTTGCATAAGTTAATGGTGTCAATACATACTCCTCATTACCCAACATGCCGGGCGTTCTTTCCAGAGGATAGGGGGTTTCTCTTTTTTTTTTTCCTTTCACTTGGCATCTCAGAGTGCATACAGAAATGACAGACAAGGTTGAACATTTTCCTTGCTTGAAGGTAAATAGTATGAATGATAGAAGACATTGATAGAAGTATTGCATAACTGATATCTAGAGCATAAAGTTCAATCAAATATTTCAATTTTCTCCTAATTTTTCTATTATTCTTCGGTTTTTTCGCGCGTAAACCCCCCCTACCCCCCCAAAACTCCTAAGATCTCTAATACTCCTGCAAACCCCCCGGAAACAGGAAAAGCTCTAGAAGTGACTTTTAGCGCTTTAGTTTATGCATGTTATATTACTTAATGTGTGTATATGCAGTAT

>DL_24 MH515173

CCGAGCTCTGCCTTCATGCAAAATACAATACATATATGTATTATCACCATTATTTTATTTCAAACATATCCTATATATAAATACATACAACTCTTAAAAAACATACACTGTTTTCCCACATATTTGTCATCAACATCTATAACTAAGAAGAACATAAACCAATAAATGAAATATTCCAATAACGATTAAATACCACTGAACGATAGTTTAAGACCGAACACAACTCTCATACAGTTAAGATATACCAAGTACCCAACATCCTATACTTCTAAATTATTTAATGTAGTAAGAGCCCACCATCAGTTGATTCCTATATGTTAACGGTTCTTGAAGGTCAAGGACAATTATTTGTGGGGGTTTCACTAATTGAATTATTCCTGGCATCTGGTTCCTATTTCAGGTCCAATAATTGTTATAATTCCCCATTCTTTCATCGACGCTTGCATAAGTTAATGGTGTCAATACATACTCCTCATTACCCAACATGCCGGGCGTTCTTTCCAGAGGATAGGGGGTTTCTCTTTTTTTTTTTCCTTTCATTTGGCATCTCAGAGTGCATACAGAAATGACAGACAAGGTTGAACATTTTCCTTGCTTGGAGGTAAATAGTATGAATGATAGAAGACATTGATAGAAGTATTGCATAACTGATATCTAGAGCATAAAGTTCAATCAAATATTTCAATTTTCTCCTAATTTTTCTATTATTCTTCGGTTTTTTCGCGCGTAAACCCCCCCTACCCCCCCAAAACTCCTAAGATCTCTAATACTCCTGCAAACCCCCCGGAAACAGGAAAAGCTCTAGAAGTGACTTTTAGCGCTTTAGTTTATGCATGTTATATTACTTAATGTGTGTATATGCAGTAT

>DL_25 MH515174

CCGAGCTCTGCCTTCATGCAAAATACAATACATATATGTATTATCACCATTATTTTATTTCAAACATATCCTATATATAAATACATACAACTCTTAAAAAACATACACTGTTTTCCCACATATTTGTCATCAACATCTATAACTAAGAAGAACATAAACCAATAAATGAAATATTCCAATAACGATTAAATACCACTGAACGATAGTTTAAGACCGAACACAACTCTCATACAGTTAAGATATACCAAGTACCCAACATCCTATACTTCTAAATTATTTAATGTAGTAAGAGCCCACCATCAGTTGATTCCTATATGTTAACGGTTCTTGAAGGTCAAGGACAATTATTTGTGGGGGTTTCACTAATTGAATTATTCCTGGCATCTGGTTCCTATTTCAGGTCCAATAATTGTTATAATTCCCCATTCTTTCATCGACGCTTGCATAAGTTAATGGTGTCAATACATACTCCTCATTACCCAACATGCCGGGCGTTCTTTCCAGAGGATAGGGGGTTTCTCTTTTTTTTTTTCCTTTCACTTGGCATCTCAGAGTGCATACAGAAATGACAGACAAGGTTGAACATTTTCCTTGCTTGGAGGTAAATAGTATGAATGATAGAAGACATTGATAGAAGTATTGCATAACTGATATCTAGAGCATAAAGTTCAATCAAATATTTCAATTTTCTCCTAATTTTTCTATTATTCTTTCGGTTTTTTCGCGCGTAAACCCCCCCTACCCCCCCAAAACTCCTAAGATCTCTAATACTCCTGCAAACCCCCCGGAAACAGGAAAAGCTCTAGAAGTGACTTTTAGCGCTTTAGTTTATGCATGTTATATTACTTAATGTGTGTATATGCAGTAT

>DL_27 MH515176

CCGAGCTCTGCCTTCATGCAAAACACAGTACATATATGTATTATCACCATTATTTTATTTCAAACATATCCTATATATAAATACATACAACTCTTAAAAAACATACACTGTTTTCCCACATATTTGTCATCAACATCTATAACTAAGAAGAACATAAACCAATAAATGAAATATTCCAATAACAATTAAATACCACTGAACGATAGTTTAAGACCGAACACAACTCTCATACAGTTAAGATATACCAAGTACCCAACATCCTATACTTCTAAATTATTTAATGTAGTAAGAGCCCACCATCAGTTGATTCCTATATGTTAACGGTTCTTGAAGGTCAAGGACAATTATTTGTGGGGGTTTCACTAATTGAATTATTCCTGGCATCTGGTTCCTATTTCAGGTTCAATAATTGTTATAATTCCCCATTCTTTCATCGACGCTTGCATAAGTTAATGGTGTCAATACATACTCCTCATTACCCAACATGCCGGGCGTTCTTTCCAGAGGATAGGGGGTTTCTCTTTTTTTTTTTCCTTTCACTTGGCATCTCAGAGTGCATACAGAAATGACAGACAAGGTTGAACATTTTCCTTGCTTGAAGGTAAATAGTATGAATGATAGAAGACATTGATAGAAGTATTGCATAACTGATATCTAGAGCATAAAGTTCAATCAAATATTTCAATTTTCTCCTAATTTTTCTATTATTCTTCGGTTTTTTCGCGCGTAAACCCCCCCTACCCCCCCAAAACTCCTAAGATCTCTAATACTCCTGCAAACCCCCCGGAAACAGGAAAAGCTCTAGAAGTGACTTTTAGCGCTTTAGTTTATGCATGTTATATTACTTAATGTGTGTATATGCAGTAT

>DL_28 MH515177

CCGAGCTCTGCCTTCATGCAAATACAATGCATATATGTATTATCACCATTATTTTATATCAAACATATCCTATATATAAATACATACTTTTTTAAAAGACATCCACTGCTCCCCCACATATTTGCCAACAACATTTACAACTAAGAGAAACATAAACCAGTAAATGGAACTTTCCAAAAACATTTCAAAACCACTAAACGACAGTTTAAGACCGAACACAACTCTCATACGTTAAGATATACCAAGTACCCACCATCCTATACTTCCGAATTATTTAATGTAGTAAGAGCCCACCATCAGTTGATTTCTCAATGTTAACGGTTCTTGAAGGTCAAGGACAATTATTTGTGGGGGTTTCACTAATTGAATTATTCCTGGCATTTGGTTCCTATTTCAGGTCCAATAATTGTTATAATTCCCCATTCTTTCATTGACGCTTGCATAAGTTAATGGTGTTAATACATACTCCTCGTTACCCACCATGCCGGGCGTTCTTTCCAGGGTGTGGGGGGTTTTTTTTTTTTTTTTCCTTTCACTTGACATTTCAGAGTGCATACAGAAAAGACAGACAAGGTTGAACATTTTCCTTGCTTGAACGGAAATAGTATGAATGGTGGTTAGATATTATTAAGAAGAATTGCATAACTGATATCTAGAGCATAAAGTTTAATCAAAATTTTAATTTTCTCCTAATTTTTCTATTAACCTTCGGTTTTTGCGCGCGTAAACCCCCCCTACCCCCCCAAAACTCCTAAGATCTCTAATATTCCTGCAAACCCCCCGGAAACAGGAAAAGCTCTAGAAGTAACTTTCAGCGCTTTAATATATGCATAAAATATTACTTAATGTGTGTATATCTAGTAC

>DL_29 MH515178

CCGAGCTCTGCCTTTGTACAAAATGCAATGCATATATGTATTATCACCATTATTCTATATCAAACATATCCTATATATAAATACATTCAATTCTTAATCAAACATAGACTGCCCCCCCACATATTTGACATCAACATTCACAACTAAGACATACATAAACCAATCAACTGAAACTTCCCAGTAAATTTGAAAAACCACTGAACGACATTTAAGACCGAACACAACCACTCATACAGTCAAGATATACCAAGTACTCAACATTCGATTCACACTCAAATATTTAATGTAGTAAGAGCCCACCATCAGTTGATTTCTTAATGTTAACGGTTCTTGAAGGTCAAGGACAATTATTCGTGGGGGTTTCACTAAATGAACTATTCCTGGCATCTGGTTCCTACTTCAGGTTCAATAATTGTTATAATCCCCCATTCTTTCATTGACGCTTGCATAAGTTAATGGTGTTAATACATACTCCTCATTACCCAACATGCCGAGCATTCTTTCCAGAGGATAGGGGGTTTCTCTTTTTTTTTTTCCTTTCATTTGGCATCTCAGAGTGCATACAGAAATGACAGACAAGGTTGAACATTTTCCTTGCTTGAAGAAAATAGTATGCGTGGTGGATAGATATTTACAGAAGAATTGCATAACTGATATCTAGAGCATAAGATTCAATCAAATATTTTAATTTTCTCCTAACTTTTCTATCAATTTTCGGTTTCTGCGCGCGTTAAACCCCCCCTACCCCCCCAAAACTCCTAAGATCTCTAATATTCCTGTAAACCCCCCCGGAAACAGGAAAAGCTCTAGAAGTGACTATCAGCGCTTTAATTTATGCATAAAATATTACTTAATGTGTGTATATATAGTAT

>DL_30 MH515179

CCGAGCTCTGCCTTCATGCAAAACACAGGACATATATGTATTATCACCATTATTTTATTTCAAACATATCCTATATATAAATACATACAACTCTTAAAAAACATACACTGTTTTCCCACATATTTGTCATCAACATCTATAACTAAGAAGAACATAAACCAATAAATGAAATATTCCAATAACAATTAAATACCACTGAACGATAGTTTAAGACCGAACACAACTCTCATACAGTTAAGATATACCAAGTACCCAACATCCTATACTTCTAAATTATTTAATGTAGTAAGAGCCCACCATCAGTTGATTCCTATATGTTAACGGTTCTTGAAGGTCAAGGACAATTATTTGTGGGGGTTTCACTAATTGAATTATTCCTGGCATCTGGTTCCTATTTCAGGTTCAATAATTGTTATAATTCCCCATTCTTTCATCGACGCTTGCATAAGTTAATGGTGTCAATACATACTCCTCATTACCCAACATGCCGGGCGTTCTTTCCAGAGGATAGGGGGTTTCTCTTTTTTTTTTTCCTTTCACTTGGCATCTCAGAGTGCATACAGAAATGACAGACAAGGTTGAACATTTTCCTTGCTTGAAGGTAAATAGTATGAATGATAGAAGACATTGATAGAAGTATTGCATAACTGATATCTAGAGCATAAAGTTCAATCAAATATTTCAATTTTCTCCTAATTTTTCTATTATTCTTCGGTTTTTTCGCGCGTAAACCCCCCCTACCCCCCCAAAACTCCTAAGATCTCTAATACTCCTGCAAACCCCCCGGAAACAGGAAAAGCTCTAGAAGTGACTTTTAGCGCTTTAGTTTATGCATGTTATATTACTTAATGTGTGTATATGCAGTAT

>DL_31 MH515180

CCGAGCTCTGCCTTTGTACAAAATGCAATGCATATATGTATTATCACCATTATTCTATATCAAACATATCCTATATATAAATACATTCAATTCTTAATTAAACATAGACTGCCCCCCCACATATTTGACATCAACATTCACAACTAAGACATACATAAACCAATCAACTGAAACTTCCCAATAAATTTAAAAGCCACTGAACGATATTTAAGACCGAACACAACCATTCATACAGTCAAGATATACCAAGTACTCAACATTCGATTCATACTCAAATATTTAATGTAGTAAGAGCCCACCATCAGTTGATTTCTTAATGTTAACGGTTCTTGAAGGTCAAGGACAATTATTTGTGGGGGTTTCACTAAATGAACTATTCCTGGCATCTGGTTCCTACTTCAGGTTCAATAATTGTTATAATTCCCCATTCTTTCATTGACGCTTGCATAAGTTAATGGTGTTAATACATACTCCTCATTACCCAACATGCCGAGCATTCTTTCCAGGGGATAGGGGGTTTCTCTTTTTTTTTTTCCTTTCATTTGGCATCTCAGAGTGCATACAGAAATGACAGACAAGGTTGAACATTTTCCTTGCTTGAGGGAAAATAGCATGCATGGTGGATAGATATTTACAGAAGAATTGCATAACTGATATCTAGAGCATAAGATTCAATCAAATATTTTAATTTTCTCCTAACTTTTCTATCAATTTTCGGTTTCTGCGCGCGTTAAACCCCCCCTACCCCCCCAAAACTCCTAAGATCTCTAATATTCCTGTAAACCCCCCGGAAACAGGAAAAGCTCTAGAAGTGACTATCAGCGCTTTAATTATGCATAAAATATTACTTAATGTGTGTATATATAGTAT

>DL_32 MH515181

CCGAGCTCTGCCTTCATGCAAAATACAATACATATATGTATTATCACCATTATTTTATTTCAAACATATCCTATATATAAATACATACAACTCTTAAAAAACATACACTGTTTTCCCACATATTTGTCATCAACATCTATAACTAAGAAGAACATAAACCAATAAATGAAATATTCCAATAATAATTAAATACCACTGAACGATAGTTTAAGACCGAACACAACTCTCATACAGTTAAGATATACCAAGTACCCAACATCCTATACTTCTAAATTATTTAATGTAGTAAGAGCCCACCATCAGTTGATTCCTATATGTTAACGGTTCTTGAAGGTCAAGGACAATTATTTGTGGGGGTTTCACTAATTGAATTATTCCTGGCATCTGGTTCCTATTTCAGGTCCAATAATTGTTATAATTCCCCATTCTTTCATCGACGCTTGCATAAGTTAATGGTGTCAATACATACTCCTCATTACCCAACATGCCGGGCGTTCTTTCCAGGGGATAGGGGGTTTCTCTTTTTTTTTTTCCTTTCACTTGGCATCTCAGAGTGCATACAGAAATGACAGACAAGGTTGAACATTTTCCTTGCTTGAAGGTAAATAGTATGAATGATAGAAGACATTGATAGAAGTATTGCATAACTGATATCTAGAGCATAAAGTTCAATCAAATATTTCAATTTTCTCCTAATTTTTCTATTATTTTTCGGTTTTTTCGCGCGTAAACCCCCCCTACCCCCCCAAAACTCCTAAGATCTCTAATACTCCTGCAAACCCCCCGGAAACAGGAAAAGCTCTAGAAGTGACTTTTAGCGCTTTAGTTTATGCATGTTATATTACTTAATGTGTGTATATGCAGTAT

>DL_34 MH515183

CCGAGCTCTGCCTTCATGCAAAATACAATACATATATGTATTATCACCATTATTTTATTTCAAACATATCCTATATATAAATACATACAACTCTTAAAAAACATACACTGTTTTCCCACATATTTGTCATCAACATCCATAACTAAGAAGAACATAAACCAATAAATGAAATATTCCAATAACGATTAAATACCACTGAACGATAGTTTAAGACCGAACACAACTCTCATACAGTTAAGATATACCAAGTACCCAACATCCTATACTTCTAAATTATTTAATGTAGTAAGAGCCCACCATCAGTTGATTCCTATATGTTAACGGTTCTTGAAGGTCAAGGACAATTATTTGTGGGGGTTTCACTAATTGAATTATTCCTGGCATCTGGTTCCTATTTCAGGTCCAATAATTGTTATAATTCCCCATTCTTTCATCGACGCTTGCATAAGTTAATGGTGTCAATACATACTCCTCATTACCCAACATGCCGGGCGTTCTTTCCAGGGGATAGGGGGTTTCTCTTTTTTTTTTTCCTTTCACTTGGCATTTCAGAGTGCATACAGAAATGACAGACAAGGTTGAACATTTTCCTTGCTTGAAGGTAAATAGTATGAATGATAGAAGACATTGATAGAAGTATTGCATAACTGATATCTAGAGCATAAAGTTCAATCAAATATTTCAATTTTCTCCTAATTTTTCTATTATTTTTCGGTTTTTTCGCGCGTAAACCCCCCCTACCCCCCCAAAACTCCTAAGATCTCTAATACTCCTGCAAACCCCCCGGAAACAGGAAAAGCTCTAGAAGTGACTTTTAGCGCTTTAGTTTATGCATGTTATATTACTTAATGTGTGTATATGCAGTAT

>DL_35 MH515184

CCGAGCTCTGCCTTCATGCAAAATACAATACATATATGTATTATCACCATTATTTTATTTCAAACATATCCTATATATAAATACATACAACTCTTAAAAAACATACACTGTTTTCCCACATATTTGTCATCAACATCTATAACTAAGAAGAACATAAACCAATAAATGAAATATTCCAACAACAATTAAATACCACTGAACGATAGTTTAAGACCGAACACAACTCTCATACAGTTAAGATATACCAAGTACCCAACATCCTATACTTCTAAATTATTTAATGTAGTAAGAGCCCACCATCAGTTGATTCCTATATGTTAACGGTTCTTGAAGGTCAAGGACAATTATTTGTGGGGGTTTCACTAATTGAATTATTCCTGGCATCTGGTTCCTATTTCAGGTCCAATAATTGTTATAATTCCCCATTCTTTCATCGACGCTTGCATAAGTTAATGGTGTCAATACATACTCCTCATTACCCAACATGCCGAGCGTTCTTTCCAGAGGATAGGGGGTTTCTCTTTTTTTTTTTCCTTTCACTTGGCATTTCAGAGTGCATACAGAAATGACAGACAAGGTTGAACATTTTCCTTGCTTGAAGGTAAATAGTATGAATGATAGAAGACATTGATAGAAGTATTGCATAACTGATATCTAGAGCATAAAGTTCAATCAAATATTTCAATTTTCTCCTAATTTTTCTATTATTCTTCGGTTTTTTCGCGCGTAAACCCCCCCTACCCCCCCAAAACTCCTAAGATCTCTAATACTCCTGCAAACCCCCCGGAAACAGGAAAAGCTCTAGAAGTGACTTTTAGCGCTTTAGTTTATGCATGTTATATTACTTAATGTGTGTATATGCAGTAT

>DL_36 MH515185

CCGAGCTCTGCCTTTGTACAAAATGCAATGCATATATGTATTATCACCATTATTTTATATCAAACATATCCTATATATAAATACATTCAATTCTTAATCAAACATAGACTGCCCCCCCACATATTTGACATCAACATTCACAACTAAGACATACATAAACCAATTAACTGAAACTTCCCAATAAATTTGAAAAACCACTGAACGACACTTAAGACCGAACACAACCATTCATACAGTCAAGATATACCAAGTACTCAACATTCGATTCACACTCAAATATTTAATGTAGTAAGAGCCCACCATCAGTTGATTTCTCAATGTTAACGGTTCTTGAAGGTCAAGGACAATTATTCGTGGGGGTTTCACTAAATGAACTATTCCTGGCATCTGGTTCCTACTTCAGGTTCAATAATTGTTATAATCCCCCATTCTTTCATTGACGCTTGCATAAGTTAATGGTGTTAATACATACTCCTCATTACCCAACATGCCGAGCATTCTTTCCAGGGGATAGGGGGTTCTCTTTTTTTTTTTCCTTTCATTTGGCATCTCAGAGTGCATACAGAAATGACAGACAAGGTTGAACATTTTCCTTGCTTAAGGAAAATAGCATGCATGGTGGATAGATATTTATAGAAGAATTGCATAACTGATATCTAGAGCATAAGATTCAATCAAATATTTTAATTTTCTCCTAACTTTTCTATCAATTTTCGGTTTCTGCGCGCGTTAAACCCCCCCTACCCCCCCAAAACTCCTAAGATCTCTAATATTCCTGTAAACCCCCCCGGAAACAGGAAAAGCTCTAGAAGTGACTATCAGCGCTTTAATTTATGCATAAAATATTACTTAATGTGTGTATATATAGTAT
